# Synaptic remodeling generates synchronous oscillations in the degenerated outer mouse retina

**DOI:** 10.3389/fncir.2014.00108

**Published:** 2014-09-05

**Authors:** Wadood Haq, Blanca Arango-Gonzalez, Eberhart Zrenner, Thomas Euler, Timm Schubert

**Affiliations:** ^1^Centre for Ophthalmology, Institute for Ophthalmic Research, University of TübingenTübingen, Germany; ^2^Werner Reichardt Centre for Integrative Neuroscience (CIN), University of TübingenTübingen, Germany; ^3^Bernstein Center for Computational Neuroscience Tübingen, University of TübingenTübingen, Germany

**Keywords:** degeneration, synapse remodeling, photopsia, retina, glutamate transporter

## Abstract

During neuronal degenerative diseases, neuronal microcircuits undergo severe structural alterations, leading to remodeling of synaptic connectivity. The functional consequences of such remodeling are mostly unknown. For instance, in mutant *rd1* mouse retina, a common model for Retinitis Pigmentosa, rod bipolar cells (RBCs) establish contacts with remnant cone photoreceptors (cones) as a consequence of rod photoreceptor cell death and the resulting lack of presynaptic input. To assess the functional connectivity in the remodeled, light-insensitive outer *rd1* retina, we recorded spontaneous population activity in retinal wholemounts using Ca^2+^ imaging and identified the participating cell types. Focusing on cones, RBCs and horizontal cells (HCs), we found that these cell types display spontaneous oscillatory activity and form synchronously active clusters. Overall activity was modulated by GABAergic inhibition from interneurons such as HCs and/or possibly interplexiform cells. Many of the activity clusters comprised both cones and RBCs. Opposite to what is expected from the intact (wild-type) cone-ON bipolar cell pathway, cone and RBC activity was positively correlated and, at least partially, mediated by glutamate transporters expressed on RBCs. Deletion of gap junctional coupling between cones reduced the number of clusters, indicating that electrical cone coupling plays a crucial role for generating the observed synchronized oscillations. In conclusion, degeneration-induced synaptic remodeling of the *rd1* retina results in a complex self-sustained outer retinal oscillatory network, that complements (and potentially modulates) the recently described inner retinal oscillatory network consisting of amacrine, bipolar and ganglion cells.

## Introduction

The mutant *rd1* mouse (Bowes et al., 1990) is an intensively studied animal model for human Retinitis Pigmentosa-related retinal degeneration. In the *rd1* retina, rod photoreceptors (rods) start degenerating around postnatal day 10 (P10) and are virtually absent by P21 (Carter-Dawson et al., 1978; Jimenez et al., 1996). During this progressive rod degeneration, cones, although not directly affected by the *rd1* mutation, undergo secondary degeneration. Some atrophied cones remain in the outer retina for over 1 year (Garcia-Fernandez et al., 1995). However, with the loss of cone outer segments after P24 (Lin et al., 2009), light-evoked retinal activity is absent in the *rd1* retina (Stasheff, 2008), thus, the disease leads to complete blindness within the first postnatal month.

Loss of light-driven activity is accompanied by a dramatic increase in spontaneous activity of the inner retina: such activity has been described in bipolar cells (Borowska et al., 2011) and ganglion cells (Margolis et al., 2008). It was suggested that AII amacrine cells and ON-cone bipolar cells form an intrinsic oscillator that serves as a potential source of this spontaneous activity (Borowska et al., 2011; Menzler and Zeck, 2011; Trenholm et al., 2012). In the healthy retina, AIIs receive glutamatergic input exclusively from rod bipolar cells (RBCs) (for review, see Bloomfield and Dacheux, 2001). However, if spontaneous activity in the AII/ON-cone bipolar cell network of *rd1* retina is intrinsic or modulated by RBCs has remained unclear.

In the outer *rd1* retina, cones and RBCs undergo structural synaptic remodeling: cones establish ectopic synapses with RBC somata (Peng et al., 2000). Rod bipolar cells lose their dendrites and down-regulate expression of metabotropic glutamate receptor 6 (mGluR6) (Strettoi and Pignatelli, 2000) as well as the respective effector cation channel TRPM1 (Krizaj et al., 2010) but remain active (Borowska et al., 2011). It is conceivable that the substantial remodeling of the *rd1* retina following photoreceptor loss leads to generation of spontaneous activity in the outer retina, which may contribute to or modulate the oscillatory activity observed in the inner retina. Investigating activity in the remodeled outer *rd1* retina is important as it improves our understanding of the general synaptic mechanisms underlying spontaneous activity in degenerated nervous tissue. Moreover, it is crucial to identify potential means for spontaneous activity suppression (Toychiev et al., 2013), which may greatly improve e.g., the responsiveness of the degenerated retina to optogenetic approaches (Lagali et al., 2008; Busskamp et al., 2010) and electronic implants (Zrenner et al., 2011; Zrenner, 2013) as treatments for vision loss.

Here, we studied neuronal activity in the outer retina of adult *rd1* mice using Ca^2+^ imaging and consecutive immunohistochemistry. We investigated how synaptic remodeling alters network function and found spontaneous, synchronized Ca^2+^ oscillations in cell clusters consisting of cones, RBCs and/or HCs. Our data suggest that gap junctionally-coupled cones, modulated by GABAergic inhibition from HCs, are responsible for generating synchronized, outer retinal activity. We further show that correlated activity between cones and RBCs depends on glutamate transporters expressed by RBCs, suggesting the appearance of atypical, sign-conserving cone-RBC synapses in the remodeled outer *rd1* retina.

## Methods and materials

### Animals

We used adult mice (both genders) at postnatal days (P) 30–60 crossbred from the transgenic *HR2.1:TN-XL* (Wei et al., 2012) and *C*x36^−/−^ (Güldenagel et al., 2001) lines with the *C3H/rd1* (Bowes et al., 1990) strain. The resulting *rd1 x HR2.1:TN-XL* and *rd1 x C*x36^−/−^ mice were homozygous for the *rd1* allele. We used *n* = 32 *rd1 x HR2.1:TN-XL* mice and *n* = 12 *C3H/rd1* mice; both lines are referred to as “*rd1*.” In addition, we used *n* = 4 *rd1 x C*x36^−/−^ mice. Animals were anesthetized with Isoflurane (Baxter, Germany) and killed by cervical dislocation. All procedures were performed in accordance with the law on animal protection (Tierschutzgesetz) issued by the German Federal Government.

### Tissue preparation

Eyes were enucleated and the retina was isolated in extracellular solution containing (in mM): 125 NaCl, 2.5 KCl, 2 CaCl_2_, 1 MgCl_2_, 1.25 NaH_2_PO_4_, 26 NaHCO_3_, and 20 glucose, and was maintained at pH 7.4 using carboxygen (95% CO_2_/5% O_2_). All chemicals were purchased from Sigma-Aldrich (Germany) and Merck (Germany). Since *rd1* degeneration progresses with age *and* retinal eccentricity, recorded fields (146 × 110 μm) were consistently taken within a ~800 μm radius from the optic disk. Retinas were incubated in extracellular solution containing 0.27 μM Fura-2-AM and 0.1% Pluronic acid (Invitrogen, Eugene, USA) for 35′ at room temperature (RT), washed, mounted on filter membranes (Millipore, 0.8 μm pores) outer retina side up, and transferred to the recording chamber, where the tissue was perfused with carboxygenated medium at 32°C.

Notably, we were not able to evoke light-driven activity in *rd1* mouse retina at P30 (as measured by Ca^2+^ imaging in the outer retina and by electrical recordings from ganglion cells; *own unpublished data*), which is consistent with earlier reports (Stasheff, 2008).

### Calcium imaging and data analysis

To record Ca^2+^ signals simultaneously in different cell types, including cones, RBCs and HCs, we decided to use a synthetic fluorescent Ca^2+^ indicator that can be loaded non-selectively into the retinal tissue (see above). Because ratiometric measurements are much less sensitive to experimental artifacts (e.g., tissue motion during drug application), we selected the Ca^2+^ indicator Fura-2 (Grynkiewicz et al., 1985). The genetically encoded Ca^2+^ sensor TN-XL, which is selectively expressed in cones in the *rd1 x HR2.1:TN-XL* strain, was here only used for the alignment of recorded with immunostained retinal regions (see Methods, Immunohistochemistry). No differences in activity were detected between cones that contained only Fura-2 (in *C3H/rd1* animals) and those containing both Fura-2 and TN-XL (in *rd1 x HR2.1:TN-XL* animals), therefore we consider it unlikely that the presence of two Ca^2+^ buffers critically affected our results.

For Fura-2 Ca^2+^ imaging, we used an upright fluorescence microscope (BX50WI, Olympus, Germany) equipped with a 40x water immersion objective (LUMPLFLN, 40x/0.80W, ∞/0, Olympus), a polychromator (POLYCHROME II, Till Photonics, Germany) and a CCD camera (TILL Imago X.Y, Till Photonics) with a resolution of 640 × 480 pixels (9.9 × 9.9 μm pixels, with a bit depth of 12), corresponding to a retinal area of 0.25 × 0.25 μm per pixel when using the 40x objective. For all experiments a 2 × 2 pixel binning was used. Single-plane two-channel image stacks of the Fura-2 fluorescence in the outer retina were acquired at 10 Hz (λ_*exc*_ = 340 and 380 nm; Olympus U-MNU filter set, 30 ms exposure time) using the TillVision software (v4.0, Till Photonics).

For offline analysis, ratio image stacks were generated by dividing the fluorescence images recorded at the two excitation wavelengths (*F*_340/380_). Cells that generated transient Ca^2+^ events (“active cells”) were identified by calculating the standard deviation (SD) image of each stack using ImageJ (http://rsbweb.nih.gov/ij). The SD images also confirmed that the observed activity was almost exclusively restricted to clearly delineated areas of 5–15 μm in diameter (cf. **Figure 2B**, left column), pointing at active cells and arguing against substantial contributions from the surrounding neuropil.

For the subsequent analysis we used custom scripts (MATLAB, The MathWorks, Germany). Active cells were manually encircled by regions of interest (ROIs) to retrieve their *F*_340/380_ (ratio) response trace. We used *F*_340/380_ as a proxy for changes in Ca^2+^ concentration. Because we were mainly interested in Ca^2+^ spiking activity (see below) and not in absolute Ca^2+^ levels, we refrained from calibrating the system. The baseline was determined by averaging *F*_340/380_ trace sections between events across the cells of each recorded field. Only cells with signal-to-noise ratios ≥ 10 (with the transient amplitude as signal and 1 SD of the baseline fluctuations as “noise”) were included in the analysis. From the ratio traces we then determined the mean Ca^2+^ event frequency (*F*_*mean*_) for each cell using a peak detection routine (based on MATLAB's findpeaks routine). In addition, the pair-wise Pearson's correlation coefficient index (*Ci*) was calculated for all cell pairs (using the corrcoef MATLAB routine). Higher event frequencies, for instance as a result of pharmacologically blocking inhibitory feedback (e.g., **Figures 6B,E**), could, in principle, result in *Ci* values purely by chance. Therefore, we tested for dependence between *F*_*mean*_ and *Ci* (using linear regression) but did not find any substantial correlations (control: *R*^2^ = 0.011; with TPMPA/Gabazine, e.g., **Figure 6B**: *R*^2^ = 0.009; with NBQX, **Figure 6E**: *R*^2^ = 0.001). This indicates that *Ci* can indeed be used as a measure for changes in correlated activity between cells. Cell position and type, as well as *F*_*mean*_ and *Ci* were then compiled to activity maps of each recorded region. The Wilcoxon signed-rank test and the student's *t*-test were used to evaluate drug effects; statistical significance is indicated as ^*^p ≤ 0.05, ^**^p ≤ 0.01, ^***^p ≤ 0.001, and all parameters are given as mean ± s.e.m.

Note that we did not detect comparable spontaneous events in cones in retinal whole mounts of *HR2.1:TN-XL* “wild-type” mice (our unpublished data). This is consistent with the observation of Borowska et al. (2011) that bipolar cells recorded in wild-type mouse whole mounts do not display spontaneous (oscillatory) activity. In contrast, in retinal slices of *HR2.1:TN-XL* “wild-type” mice we did observe spontaneous events, in particular, when HC feedback inhibition was pharmacologically blocked (see Discussion).

### Pharmacology

Drugs were bath applied for 10′ and washed out for 20′. We used (in μM): 50 TPMPA (GABA_C_ receptor antagonist; 1,2,5,6-Tetrahydropyridin-4-yl)met hylphosphinic acid), 10 Gabazine (Gz, GABA_A_ receptor antagonist; 6-Imino-3-(4-methoxyphenyl)-1(6H)-pyridazine-butanoic acid hydrobromide), 75 DL-TBOA (glutamate transporter antagonist; DL-threo-β-Benzyloxyaspartic acid) and 100 CPPG (mGluR6 antagonist; (RS)-α-Cyclopropyl-4-phosphonophenylglycine) were purchased from Tocris Bioscience; 1 strychnine (glycine receptor antagonist), 100 Verapamil (L-type voltage-gated calcium channel blocker) and 100 carbenoxolone (CBX, gap junction blocker; (3β,20β)-3-(3-Carboxy-1-oxopropoxy)-11-oxoolean-12-en-29-oic acid disodium) were purchased from Sigma-Aldrich; 100 L-AP4 (mGluR6 agonist; L-2-amino-4-phosphonobutyric acid) and 20 NBQX (AMPA/kainate-type GluR antagonist; 2,3-Dioxo-6-nitro-1,2,3,4-tetrahydro-benzo[f]quinoxaline-7-sulfonamide) were purchased from Bio Trend. For some experiments, glutamate (250 μM) was directly puffed with a glass pipette for 750 ms onto recorded outer retina neurons using a VC3−8 perfusion system (ALA Scientific Instruments, USA).

### Immunohistochemistry

After Ca^2+^ imaging, retinas were fixated with 4% paraformaldehyde (PFA) in extracellular solution for 15′ at RT and washed twice for 20′ in 0.1 M phosphate buffered saline (PBS, in mM: 20 NaH_2_PO_4_, 80 Na_2_HPO_4_, 154 NaCl; pH 7.4) at 4°C. Retinas were then incubated overnight at 4°C in blocking solution containing 0.3% Triton X-100, 1% BSA and 10% corresponding normal serum from the host animals used for generating the respective secondary antibodies. Subsequently, retinas were incubated for 1–3 days in primary antibodies (Table 1), washed 6 times for 5′ in PBS and then incubated overnight at 4°C in secondary antibodies (1:750, Alexa Fluor conjugates; Invitrogen). After mounting on slides using Vectashield (Vector, Burlingame, CA, USA) the retinas were imaged (stacks with 0.3–0.95 μm z-axis steps) using a Zeiss Imager Z1 Apotome (Oberkochen, Germany; Plan-Apochromat 5x/0.16, 20x/0.8 and EC Plan-Neofluar 40x/1.3 oil; filter set #38 for Alexa Fluor 488, #10 for Alexa Fluor 568, #50 for Alexa Fluor 633, #49 for DAPI). Fixated vertical retina sections (22 μm) were incubated overnight in primary antibodies, for 1 h in secondary antibodies and subsequently imaged as described above (stacks w/ 0.32 μm z-axis steps).

**Table 1:** **Antibodies**.

Cone photoreceptors (cones)	Chicken anti-GFP (1:200; Novus Biologicals, USA) Rabbit anti-recoverin (1:500; Chemicon International, Germany)
Rod bipolar cells (RBCs)	Rabbit anti-PKCα (1:100; Santa Cruz Biotechnology, USA) Mouse anti-PKCα (1:100; BioTrend, Switzerland)
Horizontal cells (HCs)	Mouse anti-calbindin (1:200; Swant, Switzerland)
Connexin36	Mouse anti-Cx36 (1:100; Invitrogen, USA)
EAAT5	Rabbit anti-EAAT5 (1:100; MBL, USA)

*Table listing antibodies for immunolabeling experiments*.

**Figure 1:**
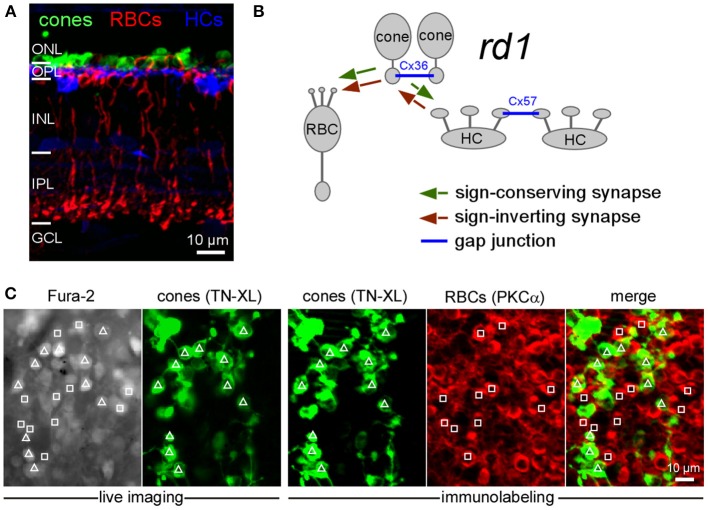
**Cell type identification following Ca^2+^ imaging in the outer retina of *rd1* mice. (A)** Vertical section of a P35 *rd1* retina immunostained for TN-XL (cones), PKCα (RBCs) and calbindin (HCs). **(B)** Drawing illustrating sign-conserving (green), sign-inverting (red), and electrical (blue) synaptic interactions in the outer *rd1* retina. For simplicity, interactions between HCs and RBCs are not shown. **(C)** From left to right: Fura-2-labeled neurons in outer retina in living P35 *rd1* wholemount retina (F_340_ channel), cones expressing TN-XL (live image taken at the same retinal location as Fura-2 image; triangles indicate cones), immunolabeling of same wholemount retina subsequent to the Ca^2+^ imaging experiment showing cones stained for TN-XL and PKCα-stained RBCs (red, squares). (ONL, outer nuclear layer; OPL, outer plexiform layer; INL, inner nuclear layer; IPL, inner plexiform layer; GCL, ganglion cell layer; RBC, rod bipolar cell; HC, horizontal cell).

In the *rd1 x HR2.1:TN-XL* mouse line, in which cones are fluorescently labeled by TN-XL, identification of cell types and alignment of Ca^2+^ imaged regions with the tissue after immunostaining was greatly facilitated. Directly after Ca^2+^ imaging, a picture of the recorded region was taken to visualize TN-XL-expressing cones (using the U-MSWG Olympus filter set). The pattern of TN-XL-positive cones was then used as landmark to find the recorded region on the immunostained retina and to determine active cell types by overlaying Fura-2 image, TN-XL image, and the images of the immunostained retina. We observed little shrinkage of the retinal tissue (5–7% compared to the living retina); in any case, because that shrinkage was homogenous we were able to register pictures from live-imaging and immunostained material reliably (in 51 of 58 cases; for examples, see Figures 1C, **7A**). In the *rd1 x HR2.1:TN-XL* line, TN-XL labeling in cones was intensified using antibodies against GFP (which also works for labeling TN-XL). In experiments using the *C3H/rd1* and *rd1 x C*x36^−/−^ lines we identified cones using recoverin staining (Lambrecht and Koch, 1992). To identify Fura-2 labeled non-cone cell types, we stained the fixed tissue for PKCα to label RBCs (Berrebi et al., 1991) and for calbindin to label HCs (Pasteels et al., 1990). The Fura-2 labeled cells that were not stained by these two antibodies likely represented cone bipolar cells.

## Results

### Neurons in the outer *rd1* retina display spontaneous activity

**Figure 2:**
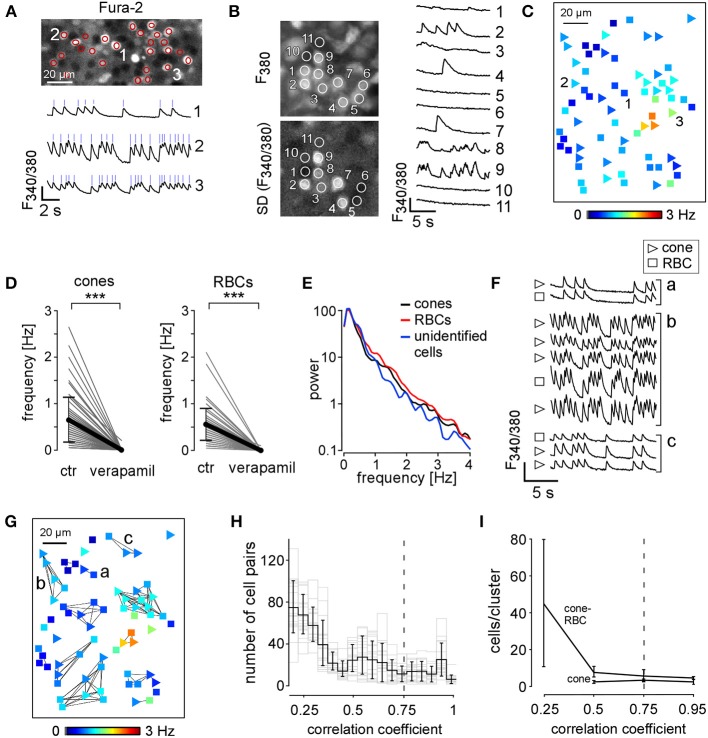
**Spontaneous activity in the outer retina of *rd1* mice**. **(A)** Fura-2-labeled *rd1* retina wholemount (SD image of *F_340/380_* ratio) with active cells encircled (regions of interest, ROIs) and exemplary Ca^2+^ traces (ROIs 1–3; blue ticks mark peaks of Ca^2+^ transients used to determine *F*_*mean*_). **(B)** left: wholemount with ROIs placed on different regions: Fura-2-labeled cells, ROIs 1–9; active cells, ROIs 2–4, 7–9; unlabeled cells/space, ROIs 10,11 (top: averaged time series, F_380_ channel; bottom: SD image of *F*_340/380_ ratio, same time series). Right: corresponding Ca^2+^ traces. **(C)** Activity map showing distribution of mean frequency (*F*_*mean*_, color-coded) of spontaneous Ca^2+^ transients in cones (triangles) and RBCs (squares). **(D)** Verapamil abolishes spontaneous activityin cones (left) and RBCs (right). (**E**) Power spectrum of the activity in cones (black, *n* = 81), RBCs (red, *n* = 108), and unidentified cells (blue, *n* = 4); data from 3 experiments. **(F)** Exemplary Ca^2+^ traces of cells exhibiting synchronous activity (clusters a-c in *G*). **(G)** Activity map showing clusters with synchronous Ca^2+^ transients (cross-correlation coefficient ≥ 0.75 indicated by lines). **(H)** Histogram showing the distribution of cross correlations between measured cell pairs (*n* = 19 retinas) for different correlation coefficients. Gray dashed line indicates correlation coefficient ≥ 0.75 used for our analysis. **(I)** Plot showing cluster size (cells/cluster) of cone-only and cone-RBC clusters for different correlation coefficients. Cluster size did not vary for correlation coefficients ≥ 0.5. All error bars indicate s.e.m.

At the adult age of mice when Ca^2+^ recordings were performed, cone somata form a single irregular cell layer in the outer *rd1* mouse retina (Figures 1A,C) (Carter-Dawson et al., 1978; Jimenez et al., 1996). Therefore, all neuron types of the *rd1* outer retina (Figure 1B, cones, HCs and bipolar cells), are accessible from the distal retinal surface and their activity can be recorded in the Fura-2 loaded *rd1* retinal wholemount using Ca^2+^ imaging (Figure 1C). Subsequent to the Ca^2+^ imaging, we performed immunocytochemistry to identify cones, RBCs and HCs (Figures 1C, **7A**). In adult *rd1* mice, up to 35% of all Fura-2 loaded cells in the outer retina were spontaneously active and displayed transient Ca^2+^ events (Figures 2A,B; cones: τ = 112 ± 182 ms, *n* = 30; RBCs: τ = 177 ± 173 ms, *n* = 22; unidentified: τ = 135 ± 152 ms, *n* = 15). While the mean frequency (*F*_*mean*_) for individual cells reached up to 3 Hz, the studied types/groups of cells were typically dominated by frequencies below 1 Hz (cones: *F*_*mean*_ = 0.65 ± 0.35 Hz, *n* = 902; RBCs: *F*_*mean*_ = 0.38 ± 0.42 Hz, *n* = 766; HCs: *F*_*mean*_ = 0.26 ± 0.15 Hz, *n* = 43; unidentified: *F*_*mean*_ = 0.57 ± 0.61 Hz, *n* = 94; see also Figure 2E).

Of each recorded field we generated an “activity map” that provided information about cell type, position, and *F*_*mean*_ of every active, identified cell (Figure 2C). Cells analyzed in *n* = 25 *rd1* retinas included 50.1% cones, 42.6% RBCs, 2.3% HCs, as well as 5% unidentified cells. The latter consisted of cells that could not be clearly assigned to stained counterparts and presumably also contained one or more types of cone bipolar cell. Because they were infrequent and likely a heterogeneous group, we did not study them further.

### Spontaneous Ca^2+^ transients in the outer *rd1* retina rely on voltage-gated Ca^2+^ channels

To assess whether spontaneous Ca^2+^ transients in *rd1* cones depend on activation of L-type voltage-gated Ca^2+^ channels (VGCCs), which are present in cones (Nachman-Clewner et al., 1999), we applied the specific antagonist verapamil. Blocking L-type VGCCs eliminated spontaneous cone activity (Figure 2D, left), suggesting that fluctuations of the cone membrane potential that activate VGGCs are required for the observed cone Ca^2+^ transients. Verapamil also eliminated spontaneous RBC activity (Figure 2D, right), likely as a result of both the blockade of L-type VGCCs in RBCs (Protti and Llano, 1998) and the reduction of glutamatergic drive from cones. Together these results suggest that the measured Ca^2+^ transients reflect electrical neuronal activity in the outer *rd1* retina. It is possible that the frequency of spontaneous Ca^2+^ events represents an underestimate of the cells' electrical activity, as we can detect only membrane potential fluctuations that are large enough to trigger VGGCs. Furthermore, it is possible that Ca^2+^ release from internal stores contributed to the measured signals (Wei et al., 2012). In principle, spontaneous activity in the inner *rd1* retina (Borowska et al., 2011) may also contribute to outer retinal activity i.e., via glycinergic and/or GABAergic input from amacrine cells (ACs) to BC terminals or possibly via GABAergic interplexiform cells to the outer plexiform layer (OPL). However, it is unlikely that the observed outer retinal activity relied on such input, because blocking glycine receptors had no effect while GABA receptor antagonists even enhanced that activity (Table 2, cf. also **Figure 6B**). Additionally, multi-electrode recordings in the *rd1* retina (Ye and Goo, 2007) showed that while spiking activity is blocked by TTX, a slow (~3.2 Hz) component persists. Because TTX was demonstrated to block inner retinal activity (Trenholm et al., 2012), the finding by Ye and Goo (2007) points at the presence of oscillatory network in the *rd1* retina that does not depend on inner retinal activity.

**Table 2:** **Pharmacology of spontaneous activity in *rd1* outer retinal neurons**.

**Cell type**	**Number of cells**	**Control (*F*_*mean*_)**	**Drug**	**Wash-out**
***rd1*, CONTROL; n = 25 RETINAS; *n* = 1711 CELLS**				
Cones	*n* = 902	0.65 ± 0.35	–	–
RBCs	*n* = 766	0.38 ± 0.42	–	–
HCs	*n* = 43	0.26 ± 0.15	–	–
***rd1*, VERAPAMIL; *n* = 3 RETINAS; *n* = 154 CELLS**				
Cones	*n* = 85	0.65 ± 0.47	0.00 ± 0.02 ^***^	0.20 ± 0.46 ^***^
RBCs	*n* = 69	0.56 ± 0.35	0.00 ± 0.01 ^***^	0.08 ± 0.21 ^***^
***rd1*, STRYCHNINE; *n* = 3 RETINAS; *n* = 160 CELLS**				
Cones	*n* = 101	0.54 ± 0.44	0.55 ± 0.33	0.51 ± 0.38
RBCs	*n* = 59	0.50 ± 0.32	0.48 ± 0.34	0.52 ± 0.30
***rd1*, TPMPA + Gz + TBOA; *n* = 6 RETINAS; *n* = 1128 CELLS**				
			TPMPA + Gz	+ TBOA
Cones	*n* = 669	0.10 ± 0.21	0.63 ± 0.58 ^***^	1.04 ± 0.63 ^***^
RBCs	*n* = 459	0.08 ± 0.21	0.62 ± 0.52 ^***^	0.41 ± 0.38 ^***^
***rd1*, L-AP4; *n* = 5 RETINAS; *n* = 293 CELLS**				
Cones	*n* = 122	0.24 ± 0.43	0.47 ± 0.60 ^***^	0.44 ± 0.50
RBCs	*n* = 171	0.55 ± 0.35	0.11 ± 0.23 ^***^	0.15 ± 0.35
***rd1*, CPPG; *n* = 5 RETINAS; *n* = 344 CELLS**				
Cones	*n* = 197	0.44 ± 0.38	0.95 ± 0.66 ^***^	0.99 ± 0.75
RBCs	*n* = 147	0.60 ± 0.43	1.07 ± 0.60 ^***^	1.05 ± 0.72
***rd1 x Cx*36^−/−^; *n* = 4 RETINAS; *n* = 265 CELLS**				
Cones	*n* = 153	0.53 ± 0.39	–	–
RBCs	*n* = 112	0.51 ± 0.42	–	–
***rd1*, TPMPA+Gz; *n* = 6 RETINAS; *n* = 499 CELLS**				
Cones	*n* = 278	0.22 ± 0.35	0.82 ± 0.48 ^***^	1.04 ± 0.70 ^***^
RBCs	*n* = 221	0.26 ± 0.37	0.92 ± 0.53 ^***^	0.99 ± 0.58 ^**^
***rd1*, NBQX; *n* = 3 RETINAS; *n* = 148 CELLS**				
Cones	*n* = 85	0.27 ± 0.47	1.14 ± 0.69 ^***^	0.61 ± 0.40 ^***^
RBCs	*n* = 63	0.40 ± 0.47	1.18 ± 0.62 ^***^	0.5 ± 0.36 ^***^
***rd1*, CBX; *n* = 3 RETINAS; *n* = 43 HCs**				
HCs	*n* = 43	0.26 ± 0.15	0.02 ± 0.11 ^***^	–

Table showing mean Ca^2+^ event frequencies (in Hz) for cones, rod bipolar cells (RBCs) and horizontal cells (HCs) for control condition, during drug application and after wash-out. Statistical significance is indicated as

** p < 0.01, and

*** *< 0.001 (control vs. drug or wash-out conditions). Gz, Gabazine; CBX, carbenoxolone*.

### Clusters of synchronous activity in the *rd1* outer retina

Calcium transients were often synchronized in neighboring cells, forming clusters of correlated oscillatory activity (Figure 2F). Such correlated activity between cells is indicated in activity maps by connecting lines visualizing synchronized cell clusters (Figure 2G). While already correlation coefficient thresholds ≥ 0.5 were sufficient to reliably identify clusters (Figures 2H,I, cf. also respective histograms in **Figures 5E, 6H,I** for different pharmacological conditions), we used a more “conservative” threshold of 0.75 to focus on the strong interactions between cells.

Most activity clusters comprised both cones and RBCs. Under control conditions, we detected in 22 *rd1* retinas a total of 144 clusters, including 92 cone-RBC clusters (5.5 ± 3.4 cells/cluster), 31 cone-only clusters (3.0 ± 1.1 cells/cluster, see Table 3) and 21 RBC-only clusters (2.2 ± 0.4 cells/cluster). It is likely that the relatively rare RBC-only clusters actually were cone-RBC clusters for which the contributing cones could not be identified—for instance because they were located outside the recorded area (see, for example, lower left cluster in Figure 2G). Therefore, we did not include RBC-only clusters in our analysis. We also found synchronous activity between cones, RBCs and unidentified cells (data not shown). Because unidentified cells were rather infrequent and represent a heterogeneous group (see above), we excluded this group from the cluster analysis. In addition, in some experiments we found synchronous oscillatory activity among HCs and between HCs and cones (**Figure 7**).

**Table 3:** **Analysis of cluster activity in the outer *rd1* retina**.

**Cluster type**	**Clusters/Field (# Clusters total)**				**Cells/Cluster**			
	**Control**		**Drug**		**Control**		**Drug**	
***rd1*, CONTROL; *n* = 22 *rd1* RETINAS; CONES *n* = 801, RBCs *n* = 707, HCs *n* = 43**								
Cone–only	1.4 ± 1 (31)		n/a		3 ± 1.1		n/a	
Cone–RBC	4.2 ± 1.7 (92)		n/a		5.5 ± 3.4		n/a	
HC-HC	1 ± 0 (3)		n/a		14.3 ± 4.1		n/a	
***rd1*, TBOA; *n* = 4 RETINAS; CONES *n* = 222, RBCs *n* = 182**								
Cone-RBC	3.75 ± 1.7 (15)		1 ± 1.4^*^ (4)		3.7 ± 1.8		2.5 ± 0.6	
***rd1*, TPMPA + Gz + TBOA; *n* = 6 RETINAS; CONES *n* = 669, RBCs *n* = 459**								
	**Control**	**TPMPA + Gz**		**TPMPA + Gz + TBOA**	**Control**	**TPMA + Gz**		**TPMPA + Gz + TBOA**
Cone–only	2.3 ± 1.6 (14)	1.5 ± 0.5 (9)		3.3 ± 2.8 (20)	2.4 ± 1.3	2 ± 0.8		2.2 ± 1.1
Cone–RBC	4.2 ± 2.9 (25)	7.8 ± 2.1^*^ (47)		3.2 ± 2.3^**^ (19)	4.7 ± 4.3	5.3 ± 4.1		3.2 ± 2.1^*^
***rd1*, L-AP4; *n* = 5 RETINAS; CONES *n* = 122, RBCs *n* = 171**								
Cone–only	1.4 ± 0.5 (7)		3.6 ± 1.5^*^ (15)		3.1 ± 1.1		2.9 ± 1	
Cone–RBC	3.8 ± 1.9 (19)		0.4 ± 0.6^*^ (3)		5.7 ± 3.5		3.4 ± 1.2	
***rd1*, CPPG; *n* = 5 RETINAS; CONES *n* = 197, RBCs *n* = 147**								
Cone–only	1.6 ± 1.2 (8)		4.2 ± 1.3^**^ (21)		2.8 ± 0.9		3.1 ± 1.2	
Cone–RBC	5 ± 1.6 (25)		1.2 ± 0.9^**^ (6)		5.6 ± 3.3		3.2 ± 1.2	
***rd1*, TPMPA + Gz + CBX; *n* = 3 RETINAS; CONES *n* = 193, RBCs *n* = 113**								
	**Control**	**TPMPA + Gz**		**TPMPA + Gz + CBX**	**Control**	**TPMA + Gz**		**TPMPA + Gz + CBX**
Cone–only	1.8 ± 0.7 (6)	3.7 ± 1.2 (11)		0 ± 0^***^ (0)	3.1 ± 1.3	3.2 ± 1.9		n/a
Cone–RBC	3.5 ± 1.8 (18)	10 ± 2.7 (30)		0.3 ± 0.6^***^ (1)	4.3 ± 1.8	4.9 ± 2.3		2 ± 0
***rd1 x Cx36^−/−^*; *n* = 4 RETINAS (7 RECORDINGS); CONES *n* = 273, RBCs *n* = 216**								
Cone–only	0.43 ± 0.53 (3)		n/a		2 ± 0		n/a	
Cone–RBC	1 ± 1.29 (7)		n/a		2.4 ± 0.7		n/a	
***rd1*, TPMPA + Gz; *n* = 6 RETINAS; CONES *n* = 278, RBCs *n* = 221**								
Cone–only	1.2 ± 0.8 (8)		2.5 ± 1.1^*^ (14)		2 ± 0		2.7 ± 1.2	
Cone–RBC	4.7 ± 1.1 (28)		8.3 ± 4.1^*^ (50)		5.5 ± 3.4		4.8 ± 4.2	
***rd1*, NBQX; *n* = 3 RETINAS; CONES *n* = 85, RBCs *n* = 63**								
Cone–only	0		2 ± 1^*^ (6)		n/a		2.2 ± 0.4	
Cone–RBC	3.3 ± 1.5 (10)		5.3 ± 2.3^*^ (16)		5.2 ± 2.4		5.2 ± 2.8	
***rd1*, CBX; *n* = 3 RETINAS; HCs *n* = 43**								
HC-HC	1 ± 0 (3)		0 ± 0^***^ (0)		14.3 ± 4.1		n/a	

Table showing cluster activity (clusters/field and cells/cluster) for cone-only clusters, cone-RBC clusters and HC-HC clusters for control and drug condition. Statistical significance is indicated as

* *p < 0.05*,

** p < 0.01, and

*** *< 0.001 (control vs. drug condition). Gz, Gabazine; CBX, carbenoxolone*.

### Cone and rod bipolar cells activity is synchronized by EAAT5 receptors

**Figure 3:**
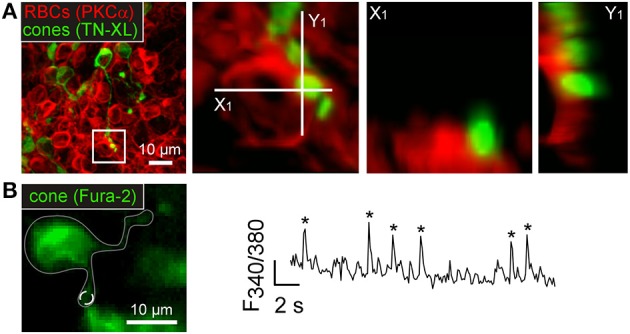
**Degenerating cones sprout and contact rod bipolar cells in *rd1* mice. (A)** Immunolabeled cones (TN-XL, green) and RBCs (PKCα, red) in P35 *rd1* retina. Higher magnification view of boxed area showing a RBC soma contacted by a cone neurite. X-images show horizontal and Y-images vertical single-plane sections of higher magnification view. **(B)** Left: Fura-2-and TN-XL-labeled cone (gray line outlines cone; dashed white circle indicates ROI). Right: Ca^2+^ trace (*F*_340/380_ ratio) with transients (indicated by asterisks) recorded in ROI in cone process (white dashed circle).

In the wild-type mammalian retina, ON bipolar cells, including RBCs, integrate glutamatergic photoreceptor output using the sign-inverting mGluR6 (Nakajima et al., 1993; Pang et al., 2010). Thus, activity in photoreceptors and ON bipolar cells is expected to be negatively correlated. Our finding of positively correlated activity between cones and RBCs in the *rd1* retina was therefore surprising. It suggests that previously described ectopic synapses between the bulb-like neurites sprouting from cones and RBC somata (Figure 3; see also Peng et al., 2000; Cuenca et al., 2004) are functional *and* sign-conserving. A potential alternative explanation is that fluorescence signals from neighboring labeled cells combined into one ROI caused false-positive correlations. However, we think that the contribution of such “bleeding-in” was marginal (see Figure 2B and Methods): in the x-y plane Ca^2+^ transients were localized to defined cell-sized areas and, thus, mostly well-separated from their active neighbors, and along the z-axis, we typically saw little “stacking” of active cells.

A possible biological explanation for sign-conserving synaptic transmission between *rd1* cones and RBCs could be degeneration-induced expression of AMPA/kainate-type GluRs on RBCs (Chua et al., 2009). However, this seems unlikely, because application of NBQX, an AMPA/kainate-type GluR antagonist, increased the activity of RBCs and the number of cone-RBC clusters (see Tables 2, 3; cf. also **Figures 6E,F**), likely due to a reduction of inhibition by HCs (for discussion, see Thoreson and Mangel, 2012), which possess AMPA/kainate-type GluRs (Schubert et al., 2006; Ströh et al., 2013).

An alternative explanation involves glutamate transporters and a transmission pathway that has been described in fish bipolar cells (Grant and Dowling, 1995). In wild-type mice, the glutamate transporter EAAT5 is present on both RBC axon terminal and soma (Wersinger et al., 2006). EAAT5 exhibits a Cl^−^ conductance that is activated by glutamate binding but independent from the actual glutamate transport across the membrane (Fairman et al., 1995; Arriza et al., 1997). Because the Cl^−^ reversal potential in the RBC dendro-somatic compartment is more positive than the resting potential (Billups and Attwell, 2002; Varela et al., 2005), activation of the EAAT5 Cl^−^ conductance by glutamate released from the cones is expected to trigger Cl^−^ efflux from the RBCs. The bipolar cells would depolarize, leading to activation of VGCCs (Protti and Llano, 1998) and, consequently synchronize Ca^2+^ transients in cones and RBCs.

**Figure 4:**
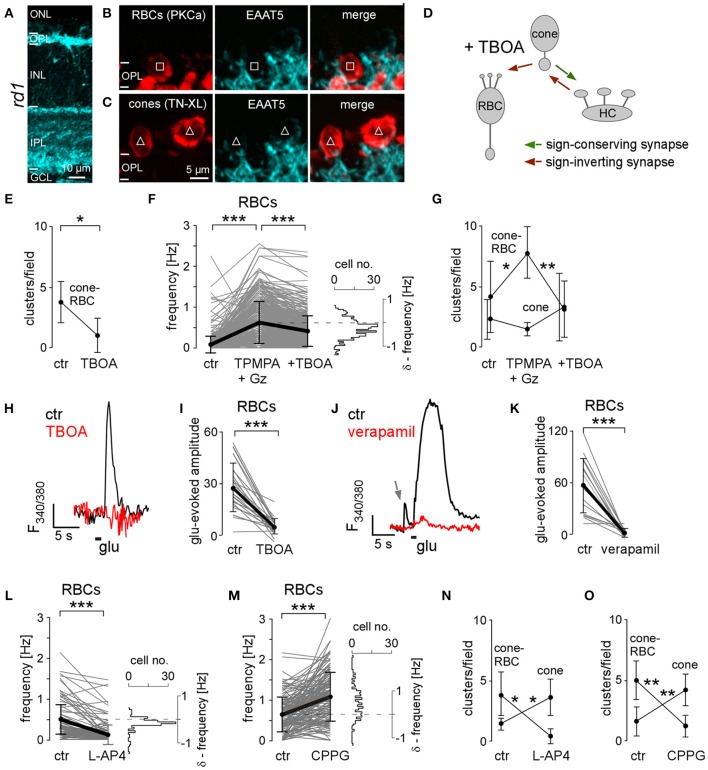
**Glutamate transporters mediate input from *rd1* cones to rod bipolar cells. (A–C)** Vertical sections of P35 *rd1* retina immunostained for EAAT5 (*A–C*, cyan) and co-labeled for RBCs (*B*, PKCα in red, square) or cones (*C*, TN-XL in red, triangles). **(D)** Drawing shows synaptic interactions with glutamate transporter antagonist TBOA blocking sign-conserved cone input to RBCs. **(E)** Number of cone-RBC clusters before (ctr) and with TBOA. **(F)** Mean frequency (*F*_*mean*_) of Ca^2+^ transients in RBCs before (ctr), with GABA receptor blockers (TPMPA+Gz), and with TBOA applied in addition (+TBOA). **(G)** Effect of the drugs (from *F*) on the number of clusters. **(H)** Example recording showing glutamate puff-evoked Ca^2+^ signal in a RBC before (ctr, black) and with TBOA (red). **(I)** Effect of TBOA on the glutamate puff-evoked Ca^2+^ signal in RBCs (*n* = 26). **(J)** Example recording showing glutamate puff-evoked Ca^2+^ signal in a RBC before (ctr, black) and with verapamil (red); gray arrow indicates spontaneous Ca^2+^ transient. **(K)** Effect of verapamil on the glutamate puff-evoked Ca^2+^ signal in RBCs (*n* = 17). **(L,M)** *F*_*mean*_ in RBCs before, with the mGluR6 agonist L-AP4 (*L*), and with the mGluR6 antagonist CPPG (*M*). **(N,O)** Effect of L-AP4 and CPPG on cluster number. All error bars indicate s.e.m.

To test this hypothesis, we first confirmed the presence of EAAT5 on *rd1* cones and RBCs using immunocytochemistry (Figures 4A–C). Then we measured the effect of the general glutamate transporter antagonist TBOA on the synchronized Ca^2+^ activity between cones and RBCs (Figure 4D). As predicted by our hypothesis, TBOA significantly reduced the numbers of cone-RBC clusters (Figure 4E; for this and further statistics, see Table 3), likely by blocking EAAT5. Because of the comparably low number of clusters that we typically found under control conditions, we also evaluated the effect of TBOA on tissue in which we first enhanced the activity (and thereby the cluster number). Blocking GABA_A/C_ receptors with the antagonists Gabazine/TPMPA increased spontaneous activity and enhanced synchronous activity between cones and RBCs (Figures 4F,G; see Tables 2, 3). This observation is consistent with inhibitory GABAergic feedback (see Horizontal cells attenuate activity in the *rd1* outer retina), as *rd1* cones (Pattnaik et al., 2000) express GABA receptors—notably, in contrast to wild-type cones (Kemmler et al., 2014). To study the TBOA effect on enhanced *rd1* activity, we first applied Gabazine/TPMPA and then added TBOA. Co-application of TBOA reduced RBC activity (Figure 4F) but at the same time increased activity in cones (Table 2). The opposite effect on the two cell types is not surprising, because—other than in RBCs (see above)—cone Cl^−^ concentration is low and, thus, the Cl^−^ reversal potential is more negative than their resting potential (Picaud et al., 1995). Hence, blockage of a transporter-mediated Cl^−^ inward current should lead to cone disinhibition. In line with the previous experiments (Figure 4E), TBOA strongly reduced the number of cone-RBC clusters also in the presence of GABA receptor antagonists (Figure 4G, Table 3). At the same time, the number of cone-only clusters increased but not significantly, which likely reflects “decoupling” of cone-RBC connections but may also partially explained by the increased number of active cones, that is, more cone-only clusters reached our correlation threshold.

The results so far predict that activation of glutamate transporters on *rd1* RBCs should cause depolarization due to their high intracellular Cl^−^ concentration and Ca^2+^ responses due to VGCC activation. To test this prediction, we puffed glutamate directly onto RBCs and found that glutamate evoked large Ca^2+^ transients (Figures 4H,J). Puffing glutamate in the presence of TBOA (Figures 4H,I) or verapamil (Figures 4J,K) failed to evoke responses, suggesting that indeed glutamate transporter-mediated depolarization and downstream activation of VGCCs underlie Ca^2+^ transients in RBCs. The possibility that glutamate acted on HCs which, in turn, synaptically excited RBCs, can be excluded: both the application of NBQX (blocking HC input) and of TPMPA+Gz (blocking potential RBC input from HCs) increased spontaneous activity in RBCs (see also below), indicating that there is no direct (GABAergic) excitatory drive from HCs to RBCs. Thus, our data indicate that correlated cone-RBC activity is based on sign-conserving ectopic synapses between cones and RBCs and mediated, at least partially, by glutamate transporters (likely EAAT5).

### mGluR modulates synaptic activity between cones and RBCs

Is there a role for mGluR6, which mediates transmission from rods to RBCs in wild-type mammalian retina (Nakajima et al., 1993), also in *rd1*? To test this, we bath-applied the group III mGluR agonist L-AP4 and antagonist CPPG and measured spontaneous activity. We found that L-AP4 decreased RBC activity, whereas CPPG led to an increase in RBC activity (Figures 4L,M, Table 2). This result is consistent with the “normal,” sign-inverting mGluR6 signaling: L-AP4 mimics glutamate binding to mGluR6, leading to the closure of downstream TRPM1 cation channels, whereas CPPG mimics glutamate unbinding from mGluR6, leading to the opening of TRPM1 channels (Morgans et al., 2009). During L-AP4 or CPPG application, cone-RBC clusters were almost absent and the number of cone-only clusters increased significantly (Figures 4N,O). This suggests that: (*i*) *rd1* RBCs still express functional mGluR6, although the transmission from the cones appears to be dominantly mediated by glutamate transporters, as we find cone-RBC activity to be positively and not negatively correlated (see previous section). (*ii*) Hyperpolarizing or depolarizing the *rd1* RBCs by modulation of the TRPM1 channels via the activation or blockade of mGluR6, respectively, appears to “clamp” the cells' membrane potential and thereby effectively “decouples” RBCs from cone input—restricting synchronous activity largely to cone-only clusters.

In addition to mGluR6 on RBCs, there is also at least one other group III mGluR in the OPL that is sensitive to L-AP4 and CPPG: the mGluR8 auto-receptor on photoreceptors (Koulen et al., 1999). Other than for RBCs, not only the antagonist CPPG significantly increased the frequency of cone Ca^2+^ transients, but also the agonist L-AP4 (see Table 2). However, the relative cone Ca^2+^ level (as reflected by the mean baseline ratio) increased with CPPG (control: 1.3 ± 0.4, CPPG: 1.7 ± 0.6, *n* = 197, *p* ≤ 0.001) but decreased with L-AP4 (control: 1.5 ± 0.6, L-AP4: 1.2 ± 0.6, *n* = 122, *p* ≤ 0.001), consistent with mGluR8 auto-receptors modulating the cone's basal Ca^2+^ level (Koulen et al., 1999). Why cones became more “spiky” despite a reduction in basal Ca^2+^ level during L-AP4 application remains to be investigated.

Taken together, glutamate released from *rd1* cones modulates both the activity of cones (auto-reception via mGluR8, EAATs) and RBCs (via mGluR6, EAATs), nevertheless, transmission from cones to RBCs appears to be dominated by sign-conserving glutamate transporter activation.

### Disrupting electrical cone coupling eliminates synchronous activity in the outer *rd1* retina

**Figure 5:**
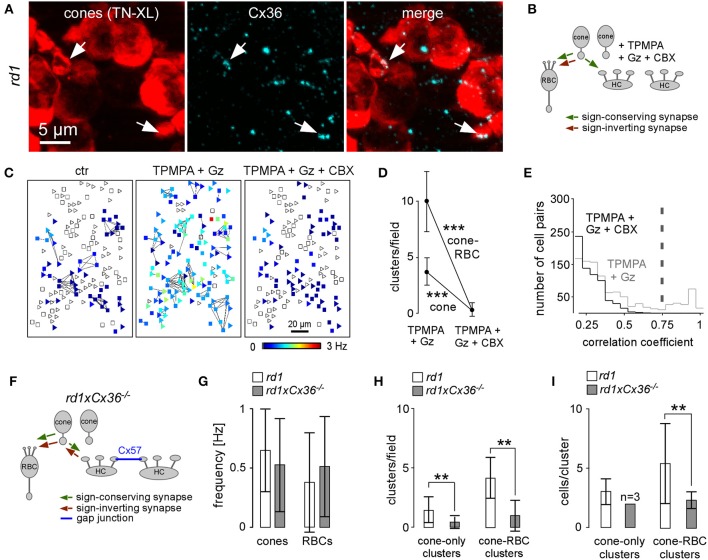
**Uncoupling of gap junctions between cones reduces correlated activity but not spontaneous activity. (A)** Left: immunolabeling of a P30 *rd1* wholemount retina showing cone somata (TN-XL, red) and smaller neurite protrusions. Middle: Cx36 immunolabeling (cyan) in same region. Right: overlay showing that cone neurite protrusions express Cx36 (arrows). **(B)** Drawing shows synaptic interactions with CBX blocking electrical coupling among cones and among HCs as well as possible ephaptic HC feedback. **(C)** Representative activity map before (ctr), with GABA receptor blockers (TPMPA+Gz), and with additional gap junction blocker CBX (cones, triangles; RBCs, squares). **(D)** Effect of the drugs on cluster number. **(E)** Distribution of correlation coefficients for control (gray line, TPMPA+Gz) and drug condition (black line, TPMPA+Gz+CBX). Gray dashed line indicates correlation coefficient ≥ 0.75 used for our analysis. **(F)** Drawing illustrating the situation in the *rd1 x C*x36^−/−^ retina with cone-cone coupling selectively abolished. **(G)** Genetic deletion of Cx36 did not affect *F*_*mean*_. **(H)** Comparison of cluster number in *rd1* and Cx36 knockout mice (*rd1 x C*x36^−/−^). **(I)** Bar graphs showing that the cone-RBC cluster size was reduced in *rd1 x Cx*36^−/−^. All error bars indicate s.e.m.

The great majority of the clusters included cones, indicating that spontaneous activity may be initially generated in the “island-like” small groups of cones and is then synaptically spread to RBCs and HCs. But what synchronizes spontaneous activity in cones in the first place? Cones are electrically coupled via gap junctions formed by connexin36 (Cx36) (Feigenspan et al., 2004). In the *rd1* retina, neuritic protrusions of remnant cones express Cx36 (Figure 5A), suggesting that clustered cones remain electrically coupled during degeneration. To uncouple the cone network, we used the gap junction blocker carbenoxolone (CBX). Since CBX is non-selective and therefore also uncouples the gap junctions of the HC network, we first reduced HC feedback using GABA receptor blockers and then co-applied CBX (Figures 5B,C). Note that CBX may also contribute to the reduction of HC-cone feedback by eliminating the alternative hemichannel-mediated ephaptic HC feedback pathway (Kamermans and Fahrenfort, 2004). We found the number of cone-only and cone-RBC clusters was significantly decreased by adding CBX (Figures 5D,E, Table 3), suggesting that electrical cone coupling is important for synchronizing *rd1* activity.

Because CBX acts non-selectively on gap junctions and hemichannels, and has potential side-effects on VGCCs (Vessey et al., 2004), we also used *rd1* mice crossbred with a Cx36 knockout line (*rd1 x C*x36^−/−^) (Figure 5F), in which photoreceptor coupling is eliminated. While the overall activity in cones and RBCs was not altered in the *rd1 x C*x36^−/−^ retina (Figure 5G, Table 2), the number of cone-only and cone-RBC clusters (Figure 5H) and the number of cells per cone-RBC clusters (Figure 5I, Table 3) were significantly lower. This supports our interpretation of the CBX effect that electrical coupling between cones is crucial for synchronizing activity clusters in the outer *rd1* retina. That the results in the *rd1* x Cx36^−/−^ retina are very similar to those with CBX also argues against a substantial suppressive effect on VGCCs at the CBX concentrations we used.

### Horizontal cells attenuate activity in the *rd1* outer retina

Mouse horizontal cells receive glutamatergic input from cones via AMPA/kainate-type GluRs (Schubert et al., 2006; Ströh et al., 2013). In turn, they provide feedback and feedforward input to photoreceptors and bipolar cells, respectively, via diverse synaptic mechanisms. These mechanisms include GABAergic, ephaptic and pH-mediated feedback (reviewed in Thoreson and Mangel, 2012). In the *rd1* retina, HC neurites sprout vertically toward the outer and inner retina (Rossi et al., 2003) and, thus, it is likely that glutamatergic transmission from cones to HCs but also feedback (and feedforward) input from HCs is altered. For example, in the wild-type mouse retina, ionotropic GABA receptors are expressed on HCs (Feigenspan and Weiler, 2004) acting as auto-receptors (Liu et al., 2013) but are not expressed on cones (Kemmler et al., 2014), whereas *rd1* cones do express GABA_A/C_ receptors (Pattnaik et al., 2000). Notably, also GABAergic interplexiform cells form synapses in the outer retina (Dedek et al., 2009) and therefore may participate in controlling the activity of neurons in the outer *rd1* retina.

**Figure 6:**
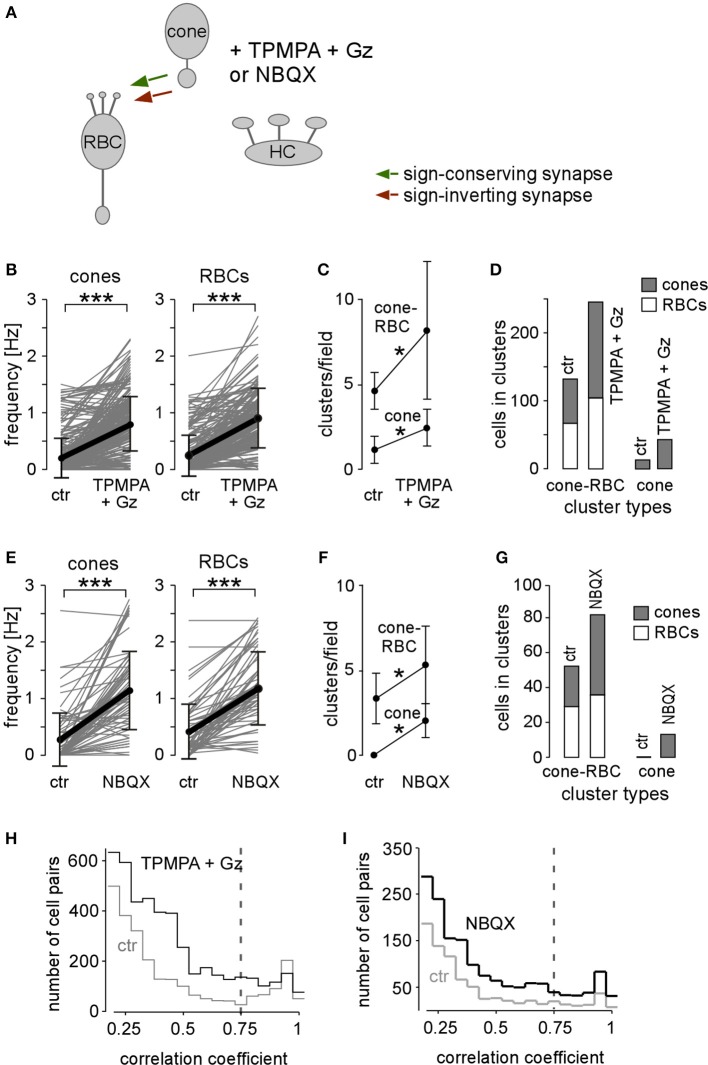
**Blocking HCs increases synchronous activity in cones and rod bipolar cells. (A)** Drawing shows synaptic interactions of outer retinal neurons with GABA receptor antagonists (TPMPA+Gz) or AMPA/kainate-type GluR antagonist (NBQX). **(B–D)** Effect of TPMPA+Gz on *F*_*mean*_ (*B*), cluster number (*C*) and cluster composition (*D*). **(E–G)** Effect of NBQX on *F*_*mean*_ (*E*), cluster number (*F*) and cluster composition (*G*). **(H,I)** Distribution of correlation coefficients for control (gray lines, ctr) and drug conditions [black lines: TPMPA+Gz (*H*), NBQX (*I*)]. Gray dashed line indicates correlation coefficient ≥ 0.75 used for our analysis. All error bars indicate s.e.m.

As shown above, GABAergic HC feedback (and potentially GABAergic inhibition provided by interplexiform cells) play a role in *rd1* outer retina in dampening cone and RBC spontaneous activity. GABA receptor blockers were particularly effective in disinhibiting cones, and likely as a consequence of increased cone input, also RBCs: in both cell types *F*_*mean*_ (Figures 6A,B, Table 2, cf. Figure 5C) and the number of all cluster types increased significantly (Figures 6C,H, Table 3). While the mean number of cells per cluster remained constant (Table 3), the cone/RBC ratio in cone-RBC clusters increased from 1 to 1.4 (Figure 6D), suggesting that HCs primarily modulate cone activity in *rd1*. The disinhibitory effect of GABA receptor blockers may be mediated either directly by acting on the GABA receptors expressed by cone axon terminals, or additionally, indirectly by inhibiting HCs and reducing ephaptic HC feedback (Liu et al., 2013). Either way, GABAergic transmission results in modulation of cone activity. Additionally, the increase of cone-RBC cluster number in the presence of GABA receptor antagonists argues against a prominent GABAergic input from the inner retina on cluster activity, but of course, cannot exclude a general modulatory effect on RBC activity.

To “silence” HCs without having to block the different possible feedback pathways to cones, we suppressed their glutamatergic input using AMPA/kainate-type GluR antagonist NBQX. Application of NBQX had similar effects as Gabazine/TPMPA: (*i*) Some cones and RBCs that were not spontaneously active under control condition started to generate Ca^2+^ transients and (*ii*) activity increased in already active cones and RBCs (Figure 6E; Table 2). (*iii*) New activity clusters formed (Figures 6F,I; Table 3), and (*iv*) the cluster composition changed, with the cone/RBC ratio in cone-RBC clusters increasing from 0.8 to 1.3 (Figure 6G) while the average number of cells per cone-RBC cluster remained unaltered (Table 3). Thus, depriving HCs from their glutamatergic input resulted in a higher number of active cones and RBCs as well as more clusters. This indicates that spontaneous activity at least in *rd1* cones is directly attenuated by HCs, in line with our GABA receptor blocker results.

It is noteworthy that NBQX does not only affect HCs, but also inner retinal activity and, thus, potentially also feedback to the OPL; i.e., via dopamine (Witkovsky, 2004). Nevertheless, we consider it unlikely that such feedback is responsible for the observed increase in cone and RBC activity, because dopamine effects are typically much slower (Witkovsky, 2004). Furthermore, the results of our glutamate puffing experiments (see Figures 4H–K) argue against the presence of ionotropic GluRs on *rd1* RBCs—in contrast to what was reported for *rd1* ON cone bipolar cells (Chua et al., 2009)—and, thus, against a direct NBQX effect on RBCs.

### Horizontal cells are functionally coupled in the *rd1* mouse retina

**Figure 7:**
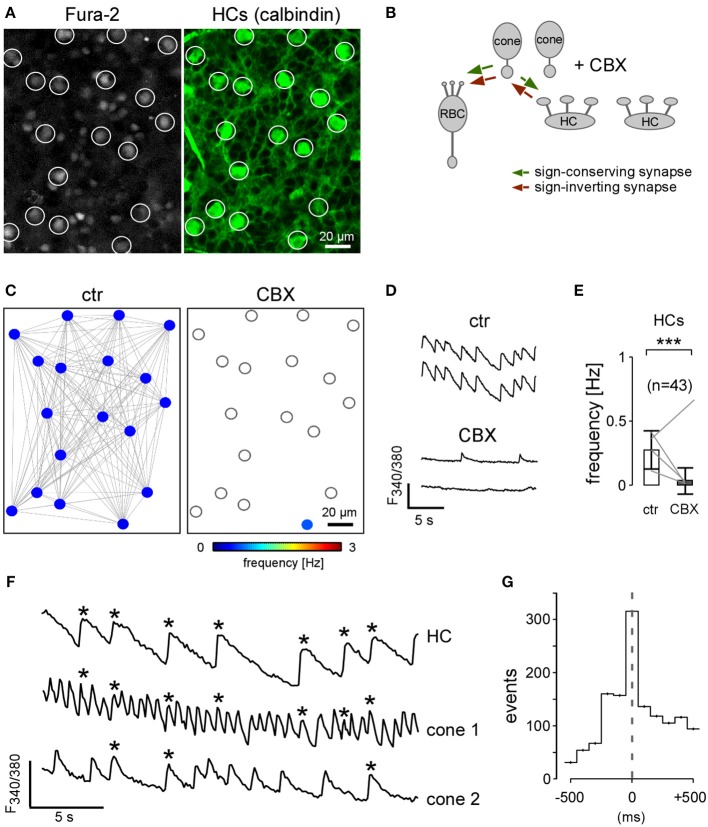
**Horizontal cells form a large electrically coupled network in the *rd1* retina. (A)** Left: Fura-2-labeled outer retinal neurons in P35 *rd1* retina (open circles mark HC cell bodies). Right: consecutive immunolabeling of the same region for calbindin confirmed that the cells are HCs. **(B)** Drawing shows that the gap junction blocker carbenoxolone (CBX) blocks coupling between HCs (and cones). **(C)** Activity map before (ctr) and during the application of CBX. **(D)** Example Ca^2+^ traces of two HCs before and with CBX. **(E)** CBX reduced *F*_*mean*_ in HCs. All error bars indicate s.e.m. **(F)** Exemplary Ca^2+^ traces of two cones and one HC (asterisks indicate synchronous Ca^2+^ transients of the HC and two neighboring cones). **(G)** Histogram showing the time-binned distribution of cone Ca^2+^ transients (*n* = 121) relative to HC events (*n* = 43; aligned to 0 ms; *n* = 3 retinas).

In addition to the cone network, also HCs form an electrical network. Fura-2 loaded HCs were easily identified because of their regularly spaced large somata, as confirmed by subsequent immunostaining with antibodies against calbindin (Figures 7A,B). In strong contrast to the fragmented cluster activity of cones and RBCs, all HCs in a recorded field participated in a single activity cluster (Figure 7C). In wild-type mice, HCs form a large electrically coupled “syncytium” using Cx57 (Hombach et al., 2004). To test if the observed activity in the *rd1* HC network was synchronized by electrical coupling, we applied CBX and found that this eliminated HC Ca^2+^ activity almost completely (Figures 7C–E, Tables 2, 3). It is unlikely that the cone input to HCs was abolished by CBX, because *selective* deletion of coupling between cones (*rd1 x C*x36^−/−^ mouse) reduced the number and size of cone-only and cone-RBC clusters but did not significantly affect *F*_*mean*_ in cones and RBCs (see above and Figures 5F–H). We therefore think that loss of HC activity was mainly due to CBX uncoupling the HC network, supporting the notion that HCs in *rd1* mice form a functional network that may spread oscillatory activity laterally in the outer retina. However, because such concerted HC activity was infrequently observed (3 out of 51 recorded fields), we did not investigate this aspect of *rd1* remodeling in detail.

### Horizontal cells and cones show synchronized activity in the outer *rd1* retina

To analyze whether HC activity is driven by cone activity or, alternatively, HCs drive cones, we measured Ca^2+^ transients and determined *F*_*mean*_ of consecutively identified HCs and cones (Figure 7G, Table 2). Typically, Ca^2+^ transients in HCs (0.26 ± 0.15 Hz) coincided with cone Ca^2+^ transients (0.53 ± 0.42 Hz), whereas not every cone transient was accompanied by a HC transient (Figure 7F). Quantification of events in the two cell types (time-binned histogram, Figure 7G) revealed that cone Ca^2+^ transients tended to precede HC events, indicating–within the limited resolution of our imaging system—that cones drive HC activity and not vice versa.

## Discussion

**Figure 8:**
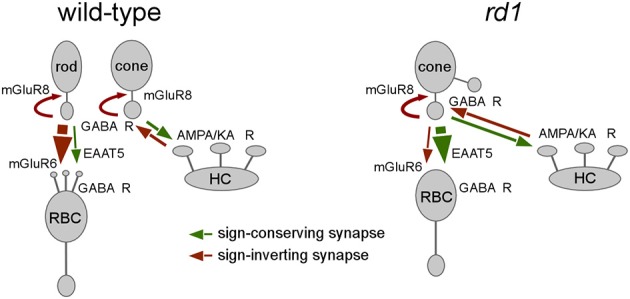
**Functional remodeling in the outer *rd1* retina**. Drawings illustrating the differences in synaptic interactions between wild-type (left) and the synaptically remodeled *rd1* (right) outer retina. For simplicity, interactions between HCs and rods/RBCs in wild-type and gap junctions are not shown (sign-conserving synapses, green arrows; sign-inverting synapses, red arrows; EAAT5, excitatory amino acid transporter 5, mGluR; metabotropic glutamate receptors; GABA R, GABA receptors; AMPA/KA R, AMPA/kainate-type glutamate receptors).

As a consequence of rod photoreceptor degeneration, the *rd1* mouse retina undergoes an extensive anatomical remodeling (reviewed in Jones and Marc, 2005), leading to spontaneous, light-independent activity in inner retinal neurons, such as ganglion cells (Stasheff, 2008). In the present study, we describe spontaneous activity in outer retinal neurons of the *rd1* mouse, and show that cones and RBCs form clusters of synchronized spontaneous activity. This correlated activity is mediated by synaptic contacts and mechanisms that result from remodeling and are atypical when compared to wild-type retina (for summary, see Figure 8): (*i*) Cones make functional synapses onto RBCs and (*ii*) signal transmission from cones to RBCs is predominantly mediated by glutamate transporters (likely EAAT5) rather than by mGluR6. (*iii*) This activity is attenuated by HC feedback, primarily via GABA_A/C_ receptors on cones rather than via a complex mechanism that involves GABA auto-receptors on HCs (Liu et al., 2013).

### Mechanisms underlying synchronous oscillatory activity in the outer *rd1* retina

Due to secondary degeneration (Carter-Dawson et al., 1978), the cone mosaic in *rd1* retina is severely disturbed, with remnant cones forming small “island-like” aggregations. Despite the grave re-organization of their synapses (e.g., Strettoi and Pignatelli, 2000), *rd1* cones express the gap junction-forming Cx36, as reported for wild-type cones (Feigenspan et al., 2004). Instead of a global gap junctionally-coupled network, *rd1* cones therefore form multiple local networks. Indeed, with cone coupling genetically ablated, activity was asynchronous, suggesting that cone coupling within clusters plays an important role in synchronizing and possibly enhancing oscillatory activity in the *rd1* outer retina. We therefore propose that spontaneous activity in the outer *rd1* retina originates in electrically coupled remnant cones—complementary to the inner retina where spontaneous activity originates in the AII/ON-cone bipolar cell network (Borowska et al., 2011; Trenholm et al., 2012).

What is the mechanism underlying intrinsic spontaneous activity in *rd1* cones? Our data show that cone activity was abolished by blocking VGCCs, indicating that fluctuations in cone membrane potential are involved. Indeed, wild-type cones were shown to possess regenerative membrane properties that can lead to spiking (reviewed in Baden et al., 2013), as demonstrated for primate cones (Schnapf et al., 1990). In mouse cones, spike-like Ca^2+^ events were also observed in recordings of vertical retina slices (Wei et al., 2012). Interestingly, while many cones showed signs of active currents (i.e., rebound Ca^2+^ transients at light-offset; cf. Figure 4 in Wei et al., 2012), only few mouse cones actually displayed spiking activity. It is tempting to compare the situation at the surface of a retinal wild-type slice with the *rd1* outer retina, since in both cases stringent control of cone output by HC feedback might be disturbed: in the slice by mechanical damage, and in *rd1* as a consequence of remodeling. In the intact retina, ON bipolar cells and HCs contact cone pedicles with invaginating contacts (Boycott and Wässle, 1999; Haverkamp et al., 2000) forming an enclosed, complex multi-synaptic structure. This well-defined structure serves to precisely control glutamate release from cones, and provides the microenvironment necessary for hemichannel-mediated ephaptic HC-cone feedback (reviewed in Kamermans and Fahrenfort, 2004), likely modulated by GABA auto-receptors on HCs (Liu et al., 2013). In *rd1*, the cone synapse is likely less “encapsulated” due to synaptic re-wiring of cone pedicles with RBCs (Peng et al., 2000), and therefore the feedback is likely less effective, leading to cone depolarization and the observed spontaneous activity, which then propagates within the local cone network. As indicated by Pattnaik et al. (2000), *rd1* cones possess GABA receptors—in contrast to the situation in wild-type (Kemmler et al., 2014)—and therefore a GABAergic HC-cone feedback is likely functional but by itself may not be sufficient to prevent spontaneous cone activity. In addition, *rd1* cones lose their outer segments and thus their source of light-evoked hyperpolarization. Consequently, the *rd1* cone membrane potential may be more depolarized than in intact cones, making it more likely that potential fluctuations reach the VGCCs' activation threshold.

### Synchronous activity in *rd1* cones and rod bipolar cells

One important finding of this study is that *rd1* RBCs receive glutamatergic cone input primarily via sign-*conserving* synapses that involve glutamate transporters (likely EAAT5). Sign-inverting mGluR6, which mediate sign-inverting input from rods to RBCs in the wild-type retina, appears to play a minor role, an observation that is in agreement with a previous study showing that *rd1* RBCs are much less sensitive for mGluR6 agonists (Chua et al., 2009). The decrease in mGluR6 on *rd1* RBCs may be related to the fact that the mGluR6 complex comprises a plethora of different membrane and cytoplasmic proteins, including the receptor itself, a G protein, nyctalopin and TRPM1 channels (Cao et al., 2011). It is therefore conceivable that mGluR6-dependent transmission is more vulnerable to degeneration-induced imbalances in protein expression level (Hauck et al., 2006)—the down-regulation of proteins such mGluR6 and TRPM1 (Strettoi and Pignatelli, 2000; Krizaj et al., 2010) may be sufficient to inactivate the mGluR6 signaling cascade. In contrast, the here described EAAT-mediated transmission pathway in *rd1* retina relies on the expression of a single trans-membrane protein, which is already present on wild-type RBCs (EAAT5, see Wersinger et al., 2006), making it a suitable candidate to dominate transmission in the newly formed, ectopic cone-RBC synapses. It is possible that the remodeled, possibly “leaky” synapse between cones and RBCs promotes an EAAT-mediated pathway, as glutamate released from the cone may diffuse further (“spill-over”) than in the wild-type situation. However, in view of the dendritic/somatic EAAT expression we found in *rd1* RBCs, action of glutamate at the distal bipolar cell end is most probable. Also, if glutamate released from cones actually were diffusing to RBC axon terminals, a hyperpolarizing response would be expected, as Cl^−^ concentration is low in the RBC terminals (Billups and Attwell, 2002; Varela et al., 2005), resulting in an EAAT-mediated Cl^−^ influx.

### Functional relevance for inner retinal networks

How does the spontaneous activity in cones and RBCs relate to the oscillations described earlier in the inner *rd1* retina? In wild-type retina, RBCs synapse onto AIIs (Bloomfield and Dacheux, 2001) and, thus, RBCs could relay the spontaneous cone activity to inner retinal neurons. While RBC axon terminals are structurally intact and AIIs are functional in *rd1* (Borowska et al., 2011), it is still unclear whether the RBC-AII synapse is functionally intact (Barhoum et al., 2008). Because blocking glutamatergic input from bipolar cells (including RBCs) failed to abolish oscillatory activity in the degenerating inner retina, it was proposed that the electrically-coupled AII/ON-cone bipolar cell network by itself is sufficient for initiating the oscillations (Borowska et al., 2011; Yee et al., 2012; Margolis et al., 2014). Additionally, the frequency of oscillatory events in *rd1* inner retinal neurons is higher (>10 Hz) than that of the Ca^2+^ signals in outer retina neurons (<3 Hz) observed in this study. This argues against the outer retina as the sole source of inner retina activity, but it does not exclude a contribution of outer retinal activity, i.e., via RBCs and possibly other bipolar cell types. Taken together, oscillatory activity in the *rd1* retina is likely generated independently by electrically coupled networks consisting of cones in the outer and AIIs/ON-cone bipolar cells in the inner retina. An important difference, however, may be that the activity in the outer *rd1* retina results from synaptic remodeling, whereas for the inner retina a correlation between aberrant activity and structural changes has not (yet) been described.

### Implication of spontaneous activity in *rd1* retina on therapeutic approaches for vision rescue

Spontaneous activity in the retina of blind human patients has not yet been directly recorded. Nevertheless, patients with retinal degenerative disorders, such as Retinitis Pigmentosa or age-related macular degeneration, often report a discomforting “misperception” of bright, light-independent flashes, a phenomenon described as photopsia (Bittner et al., 2009). It is conceivable that the spontaneous activity described in the *rd1* mouse also exists in retinal degeneration patients, potentially manifesting as photopsia. Moreover, photopsia poses a severe problem for the development of therapeutic strategies, such as electrical retinal implants (Klauke et al., 2011; Zrenner, 2013) and optogenetic approaches (Lagali et al., 2008). Recently, the implantation of electrical prostheses demonstrated partial restoration of limited vision at advanced stages of photoreceptor degeneration in humans: treated patients were able to distinguish basic geometric forms, read large letters and even discriminate facial expressions. However, some patients reported “blurring” of visual stimuli that exceeded what was to be expected from the spatial and temporal resolution of the implant chip (Zrenner et al., 2011). A possible explanation may be that signal transmission efficiency from the implant to second-order retinal neurons is reduced and degraded by spontaneous activity in the retinal network. For the same reason, optogenetic approaches that aim at rendering remnant photoreceptors or second-order neurons light-sensitive (Lagali et al., 2008), potentially suffer from spontaneous activity in the degenerating retinal network. As reported here, disrupting the electrically coupled retinal networks genetically or pharmacologically largely abolished synchronous oscillations of *rd1* outer retinal neurons. Similar effects of gap junction blockers were reported for the spontaneous activity in the inner retina (Borowska et al., 2011; Menzler and Zeck, 2011; Toychiev et al., 2013). Therefore, uncoupling electrical networks in the retina, e.g., by (more selective and, importantly, non-toxic) gap junction blockers, may possess therapeutic potential for treating photopsia.

### Conflict of interest statement

The authors declare that the research was conducted in the absence of any commercial or financial relationships that could be construed as a potential conflict of interest.
